# APRIL: A double-blind, placebo-controlled, randomized, Phase Ib/IIa clinical study of ApTOLL for the treatment of acute ischemic stroke

**DOI:** 10.3389/fneur.2023.1127585

**Published:** 2023-02-24

**Authors:** Macarena Hernández-Jiménez, Francisco Abad-Santos, Ian Cotgreave, Jaime Gallego, Bernd Jilma, Alan Flores, Tudor G. Jovin, José Vivancos, Carlos A. Molina, Joan Montaner, Joaquín Casariego, Mads Dalsgaard, María Hernández-Pérez, David S. Liebeskind, Erik Cobo, Marc Ribo

**Affiliations:** ^1^AptaTargets S.L., Madrid, Spain; ^2^Clinical Pharmacology Department, Hospital Universitario de La Princesa, Instituto de Investigación Sanitaria La Princesa (IP), Universidad Autónoma de Madrid (UAM), Madrid, Spain; ^3^Centro de Investigación Biomédica en Red de Enfermedades Hepáticas y Digestivas (CIBERehd), Instituto de Salud Carlos III, Madrid, Spain; ^4^Division of Bioeconomy and Health, Department of Chemical and Pharmaceutical Safety, Research Institutes of Sweden, Södertälje, Sweden; ^5^Neurological Center of Navarra, Navarra, Spain; ^6^Department of Clinical Pharmacology, Medical University of Vienna, Vienna, Austria; ^7^Stroke Unit, Hospital Joan XXIII, Tarragona, Spain; ^8^Cooper Neurological Institute, Camden, AR, United States; ^9^Stroke Unit, Department of Neurology, Hospital La Princesa, Madrid, Spain; ^10^Stroke Unit, Department of Neurology, Hospital Vall d'Hebron, Barcelona, Spain; ^11^Department of Neurology, Hospital Macarena, Sevilla, Spain; ^12^Aldebaran Health Intelligence, S.L, Madrid, Spain; ^13^Cureteq AG, Zug, Switzerland; ^14^Stroke Unit, Department of Neurology, Hospital Germans Trias I Pujol, Barcelona, Spain; ^15^Neurovascular Imaging Research Core, Department of Neurology, UCLA Stroke Center, Los Angeles, CA, United States; ^16^Statistics and Operations Research, Barcelona-Tech (UPC), Barcelona, Spain

**Keywords:** clinical trial, stroke, aptamer, inflammation, neuroprotection, ApTOLL, TLR4

## Abstract

In the reperfusion era, a new paradigm of treating patients with endovascular treatment (EVT) and neuroprotective drugs is emerging as a promising therapeutic option for patients with acute ischemic stroke (AIS). In this context, ApTOLL, a Toll-like receptor 4 (TLR4) antagonist with proven neuroprotective effect in preclinical models of stroke and a very good pharmacokinetic and safety profile in healthy volunteers, is a promising first-in-class aptamer with the potential to address this huge unmet need. This protocol establishes the clinical trial procedures to conduct a Phase Ib/IIa clinical study (APRIL) to assess ApTOLL tolerability, safety, pharmacokinetics, and biological effect in patients with AIS who are eligible for EVT. This will be a multicenter, double-blind, randomized, placebo-controlled, Phase Ib/IIa clinical study to evaluate the administration of ApTOLL together with EVT in patients with AIS. The study population will be composed of men and non-pregnant women with confirmed AIS with a <6h window from symptoms onset to ApTOLL/placebo administration. The trial is currently being conducted and is divided into two parts: Phase Ib and Phase IIa. In Phase Ib, 32 patients will be allocated to four dose ascending levels to select, based on safety criteria, the best two doses to be administered in the following Phase IIa in which 119 patients will be randomized to three arms of treatment (dose A, dose B, and placebo).

**Identification of the trial:**

EudraCT: 2020-002059-38 and ClinicalTrials.gov Identifier: NCT04734548 https://clinicaltrials.gov/ct2/show/NCT04734548?term=ApTOLL&cond=Stroke&draw=2&rank=1.

## 1. Introduction

Stroke is the second cause of death and the leading cause of adult disability in developed countries, affecting 17 million people worldwide each year. In the European Union, the number of patients who suffer a stroke continues to rise due to the aging of the population (1). Endovascular treatment (EVT) has improved acute stroke care over the past few years; advances in mechanical thrombectomy allow clot retrieval from large cerebral vessels with stent retrievers and aspiration catheters. Tissue plasminogen activator (rt-PA) has been the only approved specific drug for acute ischemic stroke (AIS) for two decades until 2015 (2), when the European and US guidelines also recommended mechanical thrombectomy as a first-line treatment in AIS due to a large vessel occlusion (3). In recent years, the number of patients that benefit from EVT has rapidly been growing worldwide and its indications are being expanded in selected cases to patients presenting with a large infarct core or presenting in a late time window of up to 24 h from symptom onset (3–7).

Endovascular treatment consistently achieves recanalization in 85–90% of cases. However, only approximately 15% of patients with stroke are eligible for reperfusion treatments and there is no alternative specific treatment approved for the remaining 85%. Furthermore, among those who receive reperfusion treatment, more than 50% will develop a moderate to severe disability despite successful reperfusion. In this context, new drugs with neuroprotective effects are being developed to be combined with EVT and to improve patients' outcomes after AIS since those drugs (1) may exert a protective effect directly in the area of ischemic penumbra, potentially extending the therapeutic window of reperfusion therapies, (2) limit the phenomena of hemorrhagic transformation and reperfusion damage that may occur after recanalization, and (3) effectively reach the area of damage when administered in combination with EVT (8–11).

ApTOLL, a DNA aptamer (ssDNA), is a Toll-like receptor 4 (TLR4) antagonist, a receptor that is involved in innate immune responses but also responds to tissue damage-associated molecular patterns (DAMPs), and therefore it is directly involved in a large number of diseases such as ischemic stroke. Specifically, the inflammatory component that appears in the acute phase of stroke is a very interesting target to enhance the recovery of patients, and ApTOLL displays a high therapeutic potential in this area (12–14). The efficacy of ApTOLL has been demonstrated at the preclinical level in experimental models of cerebral and myocardial ischemia (15, 16). In addition, a first-in-human study was conducted to assess the safety and pharmacokinetics of ApTOLL in healthy subjects (17). The evidence provided by both studies supports that ApTOLL could reduce the ischemic damage in several experimental models and animal species while showing an excellent safety profile in healthy humans.

In this scenario, we designed a protocol that sets the clinical trial procedures to conduct the Phase Ib/IIa clinical trial (APRIL) to assess ApTOLL safety and biological effect in patients with AIS who are eligible for EVT within 6 h to ApTOLL administration.

## 2. Methods and analysis

### 2.1. Overall design

The current version of the protocol is the APRIL V5, 11 October 2021. To design this protocol, the SPIRIT reporting guidelines (18) were used.

APRIL is a double-blind, randomized, multicenter, placebo-controlled, Phase Ib/IIa clinical Study designed to assess if the administration of ApTOLL together with EVT in AIS patients with confirmed large vessel occlusion (LVO) who are candidates to receive EVT with or without intravenous (i.v.) thrombolysis, is safe and well tolerated. The study population will be composed of men and non-pregnant women in which ApTOLL can be administered within 6 h from symptom onset. For patients with wake-up stroke, onset time will be considered as the time of symptoms first discovered.

The APRIL clinical trial will be divided into two parts: This is a Phase Ib/IIa clinical trial divided into two parts: the first part (Phase Ib) is a single dose escalation study, and the second one (Phase IIa) is an exploratory study to assess the safety and biological effect of ApTOLL at two different doses.

- Phase Ib (32 patients with AIS): a single dose, i.v. administration (30 min infusion), dose escalation with 4 single dose levels (8 patients/level), randomized (1:3), double-blind, placebo-controlled, in patients with AIS.

- Phase IIa (119 patients): a single dose, i.v. administration (30 min infusion), parallel (3 arms, placebo:ApTOLL dose A:ApTOLL dose B), randomized (√2:1:1), double-blind, placebo-controlled, in patients with AIS.

**Figure 1:**
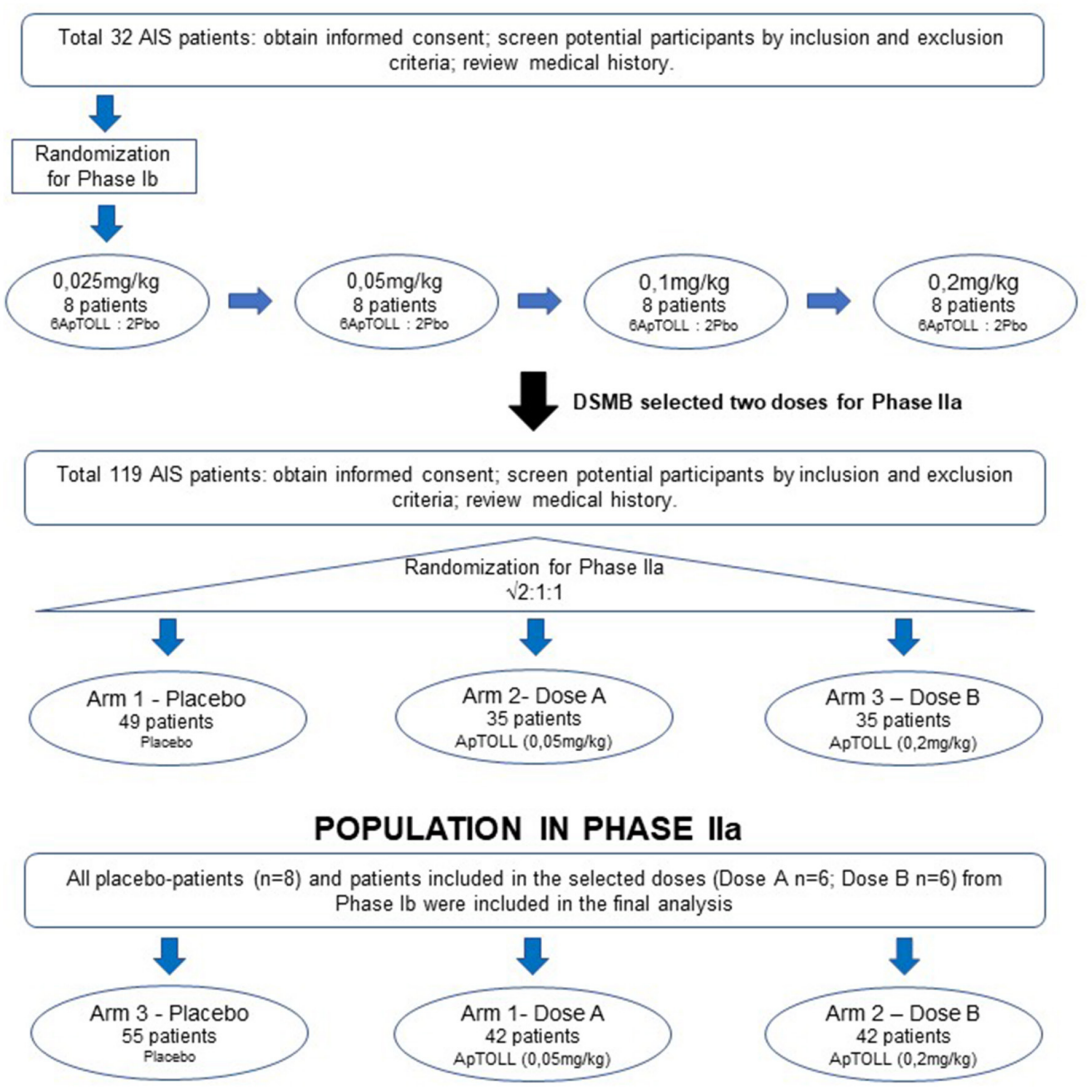
Scheme of APRIL trial. APRIL is divided into two Phases. Phase Ib is a single ascending dose where 8 patients (6 ApTOLL: 2 Placebo) will be enrolled in 4 sequential ascending dose arms (0.025, 0.05, 0.1, and 0.2 mg/kg). After analyzing safety results from Phase Ib, the DSMB will select two doses to be administered in Phase IIa. In Phase IIa three parallel arms (Dose A, Dose B, and Placebo) will be studied. At the moment of this publication, Phase Ib is completed and the doses 0.05 and 0.2mg/kg have been selected to continue during Phase IIa.

Therefore, after the completion of Phase Ib a data safety monitoring board (DSMB) unblinded to study groups will select 2 doses (A or B) to be tested in Phase IIa according to initial safety results (Figure 1).

The study population will be men and non-pregnant women with confirmed AIS with LVO in the anterior circulation, defined as per EU-US guidelines, with a <6 h window from onset of symptoms to ApTOLL administration, who are candidates to receive EVT treatment. For wake-up strokes, onset time will be considered as the time of symptoms first discovered.

**Figure 2:**
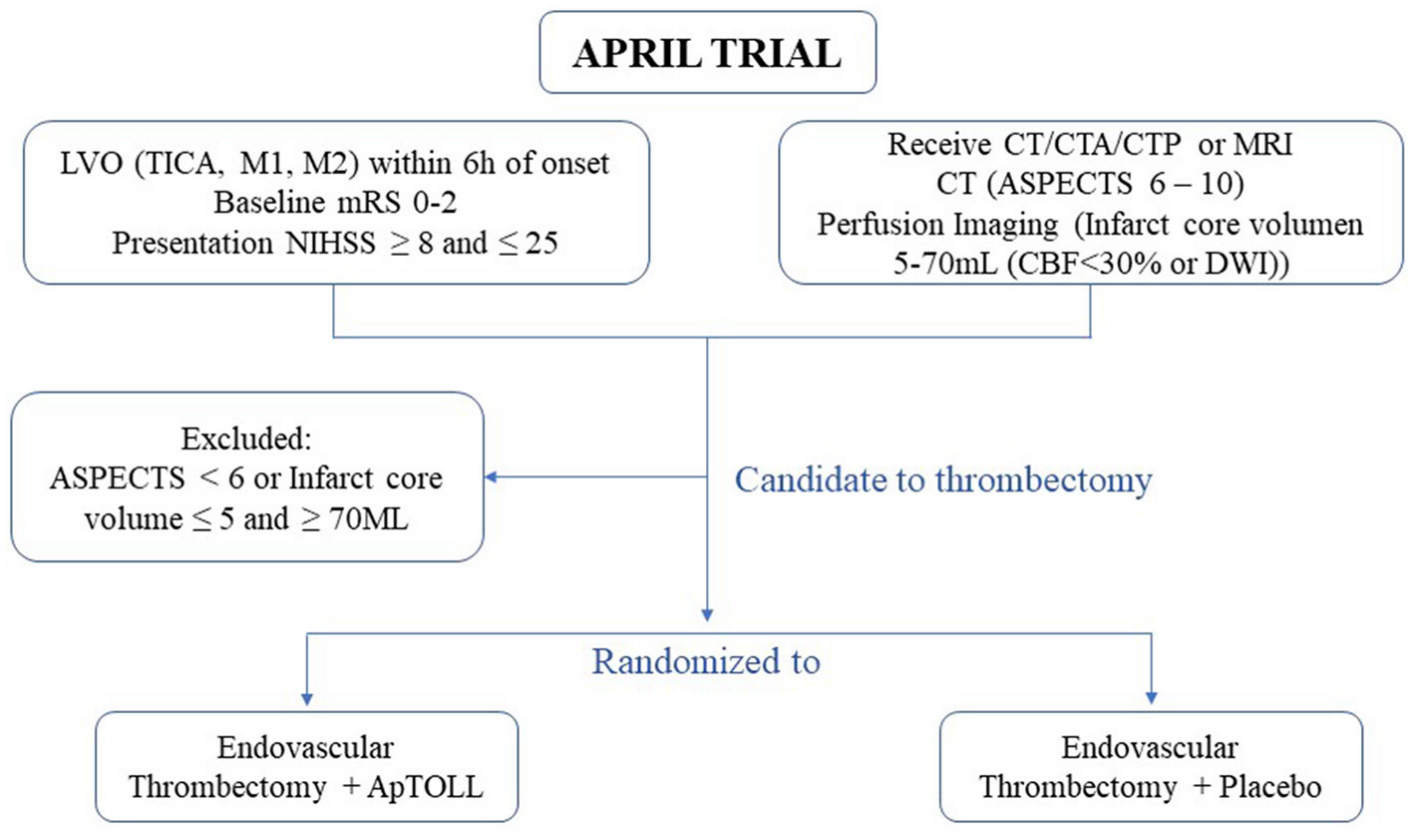
Procedures in APRIL trial. The flow of determinations to confirm stroke-related eligibility criteria in those patients enrolled in the APRIL trial.

Prior to the enrolment, all patients will undergo complete neuroimaging including non-contrast computerized tomography (NCCT), CT angiography, and CT perfusion (CTP). Only patients with an indication of EVT, based on NCCT findings (i.e., Alberta Stroke Program Early CT score, ASPECTS >5), will be evaluated as candidates for the APRIL study. Further neuroimaging inclusion criteria will be the presence of a single LVO at the level of the terminal internal carotid artery or the M1 or M2 segments of the middle cerebral artery and the identification of a favorable CTP profile as previously defined (18) to maximize the chances to identify a biological effect of the study drug. A predicted infarct core volume on CTP defined as cerebral blood flow (CBF) < 30% or diffusion-weighted imaging (DWI) between 5 and 70 ml should be identified with automated software (RAPID^®^ software) in order to be eligible for the APRIL study. After obtaining informed consent, the patients will be randomized to receive EVT + ApTOLL *vs*. EVT + placebo as shown in Figure 2. The study medication should be initiated after imaging acquisition and before EVT initiation (groin puncture). All patients will then be treated according to institutional protocols and national and European Stroke Organization (ESO) guidelines. At 90 days, the patients will undergo follow-up visit procedures.

### 2.2. Objectives and endpoints

The aim of the APRIL study is to evaluate if ApTOLL is safe and shows any biological effect in AIS patients with LVO.

- **Primary objective**: This study aimed to evaluate if the administration of ApTOLL i.v. at ascendant doses is safe and well tolerated as compared to placebo when administered with EVT in the AIS target population.
- **Secondary objectives**: Even though it is a unique study, the secondary objectives are different in the two study parts.

- Phase Ib:
  ▪ To determine the pharmacokinetic profile of ApTOLL in patients with AIS, which is evaluated by the determination of ApTOLL levels in plasma and urine.
  ▪ To select the two doses to be administered in Phase IIa according to their safety profile.
  ▪ To provide an initial estimate of the biological effect of ApTOLL on the final infarct volume (measured by magnetic resonance image—fluid attenuated inversion recovery [MRI-FLAIR] at 72±24 h) and on pro-inflammatory biomarkers linked to AIS (baseline (pre-dose) and at the end of the infusion [up to 1 h], 6, 24, 48, and 72 h post-dose).

- Phase IIa:
  ▪ To assess the biological effect of ApTOLL on the final infarct volume (measured by MRI-FLAIR at 72 ± 24 h) and on pro-inflammatory biomarkers linked to AIS (pre-dose and 6, 24, 48, and 72 h post-dose).
  ▪ To determine the biological effect of ApTOLL as measured by the functional impairment (National Institute of Health Stroke Scale [NIHSS]) at 72 h or discharge (whichever occurs first) and disability (modified Ranking Score [mRS] at 90 d post stroke).

To achieve these objectives, the following endpoints will be evaluated:

- **Primary endpoints**: Safety of ApTOLL when combined with EVT as determined by death, adverse events that occur during the study, physical examination, laboratory tests, recurrent stroke, and symptomatic intracranial hemorrhage (sICH).
- **Secondary endpoints**:

1. Mean final infarct volumes measured at 72 ± 24 h (MRI). In those cases where the MRI at 72 ± 24 h is missed, the last CT data available after ApTOLL administration should be considered.
2. Proinflammatory markers in blood between study groups.
3. Early clinical course [NIHSS; 72 h or discharge (whichever occurs first)].
4. Long-term outcome (mRS; 90 days). In those cases where the mRS 90 days is missed, the last neurological assessment available after ApTOLL administration should be considered.
5. Neuroimage proinflammatory biomarkers in MRI at 72 h and 90 d post-stroke (sub-study in some APRIL sites).

### 2.3. Inclusion and exclusion criteria

Participants will be recruited at the participating comprehensive stroke centers in Spain, France, Germany, and Portugal. All of them will have a confirmed LVO and will be candidates to receive reperfusion therapies (EVT with or without i.v. thrombolysis). Mechanical thrombectomy includes the use of any commercially available stent retrievers or aspiration catheters or combinations of the above as recommended by ESO guidelines.

Eligibility Criteria are established as follows:

#### 2.3.1. Inclusion criteria

1. Age ≥18 and ≤ 90 years.
2. Informed consent obtained from the subject or acceptable subject surrogate (i.e., next of kin or legal representative).
3. A new focal disabling neurologic deficit consistent with acute cerebral ischemia.
4. Baseline NIHSS obtained prior to randomization ≥8 points and ≤25 points.
5. Pre-stroke mRS score of 0–2.
6. Treated as soon as possible and at least within 6 h of symptom onset, defined as a point in time when the subject was last seen well (at baseline). For wake-up strokes, onset time will be considered as the time of symptoms first discovered (Treatment start is defined as study drug administration).
7. Patients should be candidates to receive EVT treatment with or without i.v. rt-PA. For such patients candidates to i.v. rt-PA therapy, rt-PA should be initiated as recommended by the European Stroke Organization (ESO) for the early management of patients with AIS, it means, as soon as possible and within 4.5 h of stroke onset (onset time is defined as the last time when the patient was witnessed to be well at baseline), with investigator verification that the subject has received/is receiving the correct i.v. rt-PA dose for the estimated weight. Should for any reason i.v. rt-PA has prematurely halted the cause and the total administered dose will be recorded. Additionally, once the patient is enrolled in the study, if recanalization is observed and documented before thrombectomy, the patient will continue in the trial and no protocol deviation will be registered.

##### 2.3.1.1. Specific neuroimaging inclusion criteria

8. Occlusion (TICI 0 or TICI 1 flow), of the terminal internal carotid artery, M1 or M2 segments of the middle cerebral artery, suitable for mechanical embolectomy, confirmed on CTA. Tandem extra-intracranial lesions may be included.
9. The following imaging criteria should also be met on admission neuroimaging:
  a) MRI criterion: volume of diffusion-weighted restriction (DWI) ≥5 and ≤70 ml determined by RAPID^®^ software OR
  b) CT criterion: Alberta Stroke program early CT score (ASPECTS) 6 to 10 on baseline CT AND infarct core determined on admission CTPerfusion by CBF<30%: ≥5 ml and ≤70 ml determined by RAPID^®^ software. NOTE: ASPECTS will be established following the investigator's criterium.
10. The subject has an indication and is planning to receive endovascular treatment for stroke according to the ESO Guidelines.

#### 2.3.2. Exclusion criteria

1. Subject has suffered a stroke in the past 1 year.
2. Occlusion (TICI 0 or TICI 1 flow) of the basilar or vertebral or posterior or anterior cerebral arteries.
3. Clinical symptoms suggestive of bilateral stroke or stroke in multiple territories.
4. Known hemorrhagic diathesis, coagulation factor deficiency, or oral anticoagulant therapy with INR >3.0.
5. Baseline platelet count of <50,000/μl.
6. Baseline blood glucose of <50 or >400 mg/dl.
7. Severe, sustained hypertension (systolic blood pressure of >185 mmHg or diastolic blood pressure of >110 mmHg). NOTE: If the blood pressure can be successfully reduced and maintained at an acceptable level using European Stroke Organization (ESO) guidelines recommended medication (including i.v. antihypertensive drips), the patient can be enrolled.
8. Serious, advanced, or terminal illness with an anticipated life expectancy of <1 year.
9. Subjects with identifiable intracerebral tumors (meningioma is considered an extracerebral tumor, so it is not included in this exclusion criterium).
10. History of life-threatening allergy (more than rash) to contrast medium.
11. Known renal insufficiency with creatinine ≥3 mg/dl or glomerular filtration rate (GFR) of <30 ml/min.
12. Cerebral vasculitis.
13. Evidence of active systemic infection.
14. Known current use of cocaine at the time of treatment.
15. Patient participating in a study involving an investigational drug or device that would impact this study.
16. Patients that are unlikely to be available for a 90-day follow-up (e.g., no fixed home address, a visitor from overseas).
17. Female who is pregnant or lactating or has a positive pregnancy test at the time of admission.

##### 2.3.2.1. Specific neuroimaging exclusion criteria

18. CT or MRI evidence of hemorrhage (the presence of microbleeds is allowed).
19. Significant mass effect with midline shift.
20. Suspicion of aortic dissection presumed septic embolus or suspicion of bacterial endocarditis.

### 2.4. Enrollment and randomization

Before starting any procedure, patients or their legal representatives will receive oral and written information about the study (objectives, risks, and benefits) and will provide their informed consent.

All patients who meet the eligibility criteria will be eligible for APRIL clinical trial enrollment. This will include both patients who are directly admitted to the study site and patients who are transferred from an external hospital (*drip and ship*). The time of enrollment is the time when the patient is randomized (through an electronic case report form [eCRF]). An automated date and time are generated by the platform. Informed consent must be signed by the patient or their authorized legal representative before any study specific treatment is performed.

In both Phase Ib and IIa parts of the trial, randomization will be done prior to the study treatment.

#### 2.4.1. Treatment blinding

The study is masked in a double-blind fashion, i.e., neither the patient nor treating physicians will be aware of the administered treatment. The solution of ApTOLL or matching placebo is limpid, transparent, and colorless.

#### 2.4.2. Enrollment and randomization Phase Ib

During Phase Ib, four ascending dose levels (0.025, 0.05, 0.1, and 0.2 mg/kg) will be completed. Within each dose level group, 8 patients will be randomized to receive ApTOLL plus EVT *vs*. Placebo plus EVT at a 3:1 ratio.

A staggered dosing scheme will be used to maximize the safety of the patients. After each dose level (i.e., once performed the follow up visit at 72 h of the last patient included in each dose level), the DSMB will evaluate the safety results and will approve the next dose level, if appropriate. The safety parameters to be evaluated are (1) any suspected serious adverse reactions (SUSAR), serious adverse event (SAE), or AE that could be related to the drug administration and (2) blood biochemistry parameters.

#### 2.4.3. Transition from Phase Ib to Phase IIa

Before starting Phase IIa part of the study, and once safety data from the LPI in Phase Ib (SAD) is obtained (follow up 72 h visit from LPI last level), whole safety results will be evaluated to choose the most appropriate doses to be studied in Phase IIa part. The DSMB will evaluate the following safety parameters to select the two optimal doses to be studied in Phase IIa (Dose A, Dose B):

1. Regarding safety parameters in the stroke field, special attention will be paid to the occurrence of

- Death: mortality data will be carefully collected for underlying causes.
- Intracranial hemorrhage (ICH) and hemorrhagic transformation (HT) result in new symptoms or worsening of the existing ones. ICH refers to any bleeding within the intracranial vault, including the brain parenchyma and surrounding meningeal spaces. HT represents the conversion of a stroke into an area of hemorrhage.
- Brain edema resulting in herniation and neurological worsening or death.
- Further potential AEs related to stroke to be carefully documented include the following:
  - Epileptic seizures.
  - Cardiac conduction disturbances, arrhythmias.
  - Effects on coagulation and fibrinolysis.
  - Hypotension/hypertension.
  - Hyperglycemia.
  - Hyperthermia.
  - Severe infections.
  - Deep vein thrombosis, pulmonary embolism, and venous thromboembolism.
  - Vomiting.
  - Anxiety, hallucinations, and agitation.

2. Regarding safety parameters related to the drug, special attention will be taken in relation to the following:

- Complement activation: CH50 levels (50% hemolytic complement) and C3/C4 levels (Complement factors).
- Biochemistry: CK (Creatine Kinase) and CRP (C-Reactive Protein).
- Coagulation parameters: activated Partial Thromboplastin Time (aPTT), Prothrombin activity (PT), and International Normalized Ratio (INR).

#### 2.4.4. Enrollment and randomization in Phase IIa

In Phase IIa, three arms will be studied (Placebo, and ApTOLL at dose A or B). Eligible patients will be randomized into placebo plus EVT *vs*. ApTOLL (dose A) plus EVT *vs*. ApTOLL (dose B) plus EVT, at a **√**2:1:1 ratio which, in turn, yields a probability of random assignment of 0.41, 0.29, and 0.29, respectively.

In Phase IIa, the DSMB will analyze AEs and SAEs when approximately 20 patients have been included in each treatment group and all of them have reached the 72 h follow-up visit.

In both Phase Ib and Phase IIa, the Clinical Adjudication Committee (CAC) will analyze all AEs and SAEs to determine which ones are related to the medication and will communicate this decision to the DSMB. With this information, DSMB will review the reported AEs and SAEs.

### 2.5. Study treatments

Both treatments (ApTOLL and placebo) will be administered intravenously using an infusion pump for 30 min.

#### 2.5.1. Study treatment

ApTOLL and placebo are formulated as a powder for concentrate for solution for infusion. The study is prepared at the required dose, following the applicable dose level, just before the administration. The placebo treatment matches the appearance of ApTOLL when as a powder for concentrate for solution for infusion and when reconstituted.

ApTOLL (7 mg) or placebo must be reconstituted in all cases with 3 ml of water for injections (stock solution). The milligrams to be injected should be calculated based on the patient's weight. Then, the corresponding milliliters should be taken from the stock solution and introduced in the saline bag (100 ml of sodium chloride 9 mg/ml (0.9%) solution for injection).

The administration of ApTOLL/placebo should be started before EVT is initiated (before groin puncture).

To establish the dosing scheme to be implemented in the APRIL study, the ApTOLL-FIH-01 study and body surface extrapolation from experimental models have been considered. In the ApTOLL-FIH-01 study, seven dose levels in healthy volunteers were assessed (from 0.01 to 1 mg/kg of ApTOLL) and no adverse reactions were detected at any dose. The pharmacokinetics observed in the subjects showed that maximum C_max_ and exposure (AUC) are reached at 0.2 mg/kg and remain stable despite increasing doses (17). This observation provides the rationale for the highest dose currently chosen in APRIL. Regarding the body surface extrapolation, the therapeutic dose confirmed in rats was 0.45 mg/kg so the estimated therapeutic dose in humans would be 0.07 mg/kg. Therefore, the starting dose for APRIL Phase Ib will be 0.025 mg/kg (0.2 with a safety factor of 8) and the dosage scheme for Phase Ib will be as follows (Table 1).

**Table 1:** Dose escalation in Phase Ib.

**Dose level**	**Dose of ApTOLL (mg/kg)**	**Number of patients to be enrolled (Ratio ApTOLL: placebo)**
1	0.025	8 (6 ApTOLL: 2placebo)
2	0.05	8 (6 ApTOLL: 2placebo)
3	0.1	8 (6 ApTOLL: 2placebo)
4	0.2	8 (6 ApTOLL: 2placebo)

#### 2.5.2. Medications/treatment permitted and not permitted before and during the clinical study

In addition to the study drug, patients will be treated according to the ESO guidelines.

### 2.6. Study assessments and procedures

**Figure 3:**
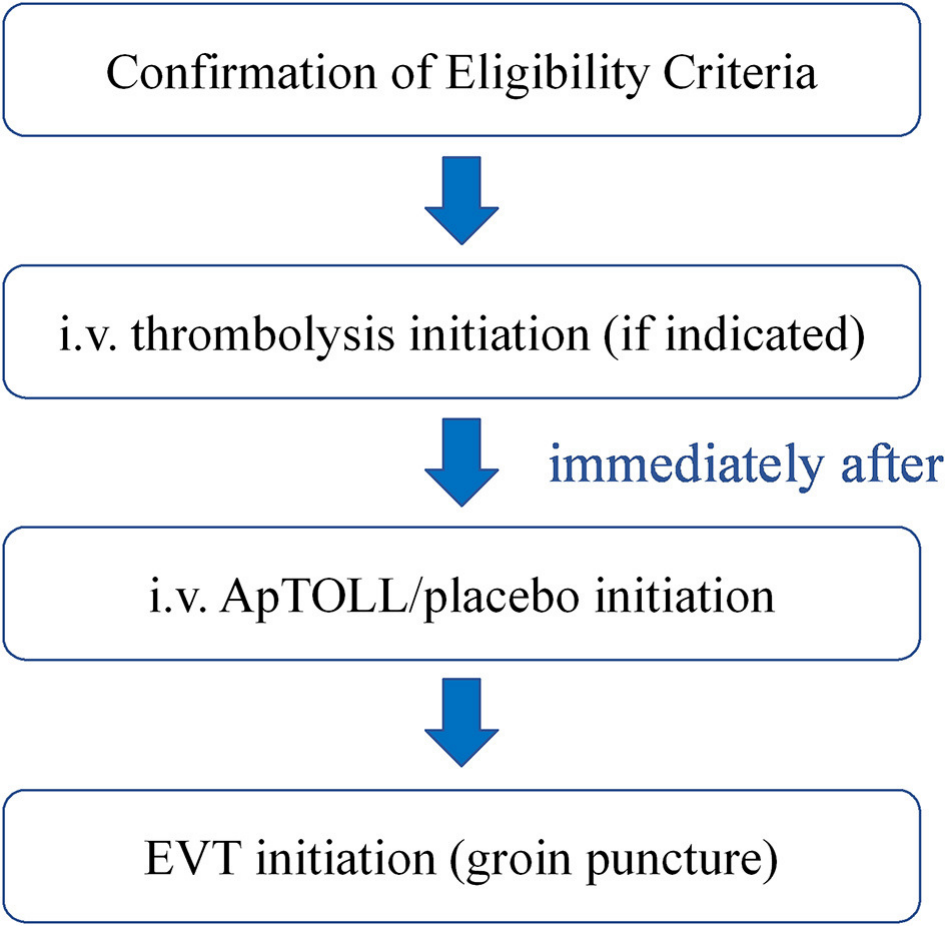
Flow diagram of the treatment in the APRIL trial. After confirmation of the eligibility criteria, intravenous thrombolysis will be initiated in those patients where indicated. Then, ApTOLL/placebo infusion will start, and the groin puncture will be performed immediately after.

The flow of treatment should be the following (Figure 3).

Each subject will undergo the following procedures (Table 2).

- **Informed consent form signature:** prior to the dosing, patients and/or legal representatives must receive oral and written information concerning the study design, objectives, and possible risks that may arise from their inclusion in the study. If they subsequently agree to participate, they or their legal representatives must sign the informed consent form, under the awareness of their ability to withdraw from the study at any time and for any reason.
- **Collection of previous clinical history**: the relevant patient's medical history, as well as substance use (toxic habits) and previous concomitant medication, should be collected.
- **Admission details**: time of symptoms onset or last time seen well (LTSW) and the time the patient arrives at the hospital should be collected.
- **Demographics and physical examination**: including age, gender, race, height, and body weight should be collected.
- **Vital signs determination**: blood pressure (BP), heart rate (HR), and body temperature (BT) are measured before the administration of study medication and during hospitalization.
- **Pre-stroke and follow-up mRS and pre-randomization NIHSS assessment**.
- **Pregnancy test (in women with childbearing potential)**.
- **Determinations of hemogram, coagulation, and biochemistry**: blood samples will be analyzed locally at every hospital participating in the clinical trial to determine hemogram (red blood cells, hemoglobin, hematocrit, mean corpuscular volume, platelets, leukocytes, and leukocyte formula), coagulation [INR, prothrombin activity (PT) and activated Partial Thromboplastin Time (aPTT)], and biochemistry [GOT, GPT, LDH, alkaline phosphatase, GGT, total bilirubin, uric acid, creatinine, urea, glycemia, cholesterol, triglycerides, albumin, Na+, K+, creatine kinase (CK), and C-reactive protein (CRP)].
- **Blood test to determine complement activation:** CH50 (50% hemolytic complement) and C3/C4 (complement factors).
- **ECG monitoring:** cardiac rhythm monitoring is performed during EVT and at least during the following 48 h. Only clinically significant abnormalities will be collected, as adverse events.
- **Adverse events assessment**.
- **Monitorization of study medication site of injection for side effects**.
- **Endovascular thrombectomy:** EVT will be initiated (groin puncture) after randomization and starting infusion of the study drug. EVT should be initiated within 8 h from symptoms onset (ApTOLL should be administered within 6 h after stroke onset). Individual investigators may use any approved device or any combination of devices to remove thrombus from the TICA, MCA M1 segment, or, if needed, from M2 segments of the intracranial circulation. Standard medical therapy, based on current ESO guidelines, will also be provided for all patients.
- **Imaging procedures**: non-contrast CT, CTA, CTP, and MRI. CT images will be read by appropriately trained local clinicians. ASPECTS for patient selection will be determined independently by the local clinicians. CTP images with mismatch determination will be read by iSchemaView automated RAPID software. All brain imaging from stroke onset through hospital discharge, including the MRI, CTP/MRP, and CT, as well as angiographic images, obtained for the diagnostic and therapeutic portions of the procedure, will be assessed independently of each other and blinded to treatment allocation at the APRIL Central Imaging Core Lab (ICL).
- **Neuroimage sub-study** (in Phase IIa and in some Spanish) centers, a second MRI will be performed at day 90 ± 14 d post-procedure.
- **Fibrinolytic therapy:** Patients will receive standard ESO guidelines directed medical therapy, which can include i.v. Alteplase/Tenecteplase administration in patients presenting within the first 4.5 h from LTSW and meeting other ESO label criteria. Special attention will be given to the possibility of existing drug interactions between rtPA and ApTOLL, both in pharmacokinetics and primary/secondary endpoints analyses. Post-thrombolysis patients will be treated based on standard study site protocols for these patients.
- **Proinflammatory biomarkers determination:** analysis of proinflammatory biomarkers is done at pre-dose and 6, 24, 48, and 72 h post-dose. At each timepoint, approximately 9 ml of whole blood will be collected into a syringe and divided into two tubes for plasma and serum isolation. Proinflammatory biomarkers to be tested will be TLR4, IL6, INFγ, TNFα, CXCL10, copeptin, and serum amyloid a (SAA).
- **Pharmacokinetics (only in Phase Ib part of the study):** blood samples for pharmacokinetic assessment, included in Phase Ib part of the study, will be collected according to standard operating procedures recommended by the analytical laboratory. In all subjects from Phase Ib, six blood samples will be taken at the following times: pre-dose, and at the end of the infusion (up to 1 h), 6, 24, 48, and 72 h (±30 min) from the end of the infusion.
- **Urine analysis for elimination assessment (only in Phase Ib part of the study):** urine samples will be taken to detect ApTOLL. When possible, every urine sample from every patient, after study drug administration and up to the follow up 24 h post-random or discharge (whatever occurs first) will be collected according to standard operating procedures recommended by the analytical laboratory.

**Table 2:** Schedule of events.

**Assessments**	**Baseline information–study allocation**	**Thrombectomy**	**Follow up 24 h**	**Follow up 48 h**	**Follow up 72 h**	**Follow up 90 days**
Admission details	X					
Demographics and physical examination	X					
Medical history and baseline medication	X					
Eligibility criteria	X					
Informed consent	X					
Vital sings (BP, HR, BT)	X		X	X	X	
mRS	X				X	X
NIHSS	X		X	X	2X^1^	X
Pregnancy test	X					
Randomization	X					
Blinded study medication administration	X					
Monitorization of study medication site of injection	X	X	X	X	X	
Blood test: *Hematology and biochemistry including INR*	X		X			
Blood test: *aPTT, PCR and complement activation*	X		X	X	X	
Proinflammatory biomarkers in blood (Phase Ib)	3X^2^		X^2^	X^2^	X^2^	
Proinflammatory biomarkers (Phase IIa)	2X^2^		X^2^	X^2^	X^2^	
Pharmacokinetics (in Phase Ib)	3X^3^		X^3^	X^3^	X^3^	
Urine analysis for elimination assessment (in Phase Ib)		X^4^	X^4^			
ECG	X^5^	X	X	X		
Non-contrast CT	X		X			
CTA	X					
CT-P	X					
DWI-MRI	X^6^					
FLAIR-MRI					X	X^7^
EVT details^8^		X				
Angiogram		X				
Stroke etiology						X^9^
EuroQol EQ-5D						X
Relevant meds	X	X	X	X	X	X
Adverse events	X	X	X	X	X	X

X^1^, NIHSS should also be performed on day 5 or discharge (whatever occurs first).

X^2^, In Phase Ib, proinflammatory biomarker samples should be taken at pre-dose, and at the end of the infusion (up to 1 h), 6, 24, 48, and 72 h post-dose. In Phase IIa, proinflammatory biomarkers will be analyzed at pre-dose and 6, 24, 48, and 72 h post-dose.

X^3^, Pharmacokinetic samples should be taken at pre-dose, and at the end of infusion (up to 1 h), 6, 24, 48, and 72 h from the end of infusion.

X^4^, Collect, if possible, urine samples from the study drug administration for 24 h.

X^5^, First ECG should be performed at 6 h post-dose.

X^6^, Included only in those hospitals where the inclusion is performed based on MRI criteria (i.e., France).

X^7^, Only for sites participating in image sub-study.

EVT^8^, Groin puncture should be done before 8 h after symptoms onset. For wake-up strokes, onset time will be considered as the time of symptoms first discovered.

X^9^, Stroke etiology will be completed when available.

### 2.7. Statistical analysis

A statistical analysis plan (SAP) including the list of all tables, listings, and graphs will be issued before the study database lock.

The analysis will assume a progressive (monotonicity) relationship (i.e., any dose above an unsafe dose will also be considered unsafe).

Those patients enrolled in Phase Ib who received a placebo or the selected doses in phase Ib will be combined with Phase IIa patients to increase statistical power. To successfully combine the patients from both study phases, the time between phases is minimal, and the design, the follow-up, and data collection will be strictly the same in Phase Ib and Phase IIa parts of the trial, with the only exception of sequential blood and urine sampling for pharmacokinetics and pharmacodynamics performed only in Phase Ib.

Categorical variables will be summarized with counts and percentages. Continuous variables will be summarized with the mean, the standard deviation, the median, the inter-quartile range, the minimum, and the maximum.

Patient's compliance with eligibility criteria and treatment administration, major protocol non-compliances, patient's withdrawals and the reason for withdrawal (e.g., AE, protocol non-compliance, lost to follow up, failed to return, consent withdrawal, and other reasons) and assignment to each analysis population will be summarized by means of the appropriate statistics.

The main results will be provided with 90% confidence intervals.

## 3. Discussion

Stroke is a devastating and debilitating disease with limited therapeutic options. To date, European and US guidelines for stroke treatment only recommend the use of thrombolytics and/or EVT. However, revascularization therapies alone appear to be insufficient for a substantial proportion of patients, and the combination of recanalization with neuroprotectants is emerging as a promising strategy to improve the outcomes of patients who suffer a stroke (8–11). This possibility to reestablish cerebral flow with EVT generates a new scenario for testing drugs with potential neuroprotective effects. In fact, Nerinetide (NA-1), a neuroprotectant focused on the inhibition of PSD-95 implicated in the excitotoxic process developed after stroke, has been shown to improve outcomes in a subpopulation of patients with AIS when administered in combination with EVT [ESCAPE-NA1 trial (19)].

In this context, we propose the APRIL clinical trial protocol (EudraCT: 2020-002059-38), a clinical study to assess the safety and biological effect of ApTOLL, an anti-inflammatory drug with proven neuroprotective effects in preclinical models. The study will focus on patients with AIS who are candidates for EVT (with or without thrombolysis) within 6 h from stroke symptoms onset to ApTOLL administration with the objective of ensuring recanalization.

Patients undergoing EVT replicate transient ischemic models and are therefore ideal candidates for neuroprotection studies since the expected recanalization rates are around 85-90% offering a homogeneous population with measurable infarct core, tissue at risk, and occlusion location that will be recanalized in most cases within few hours after admission. The profile of the patient candidates for EVT however might be different in the early (0–6 h) vs. the late (6–24 h) time window and, also, there are patients who still show salvageable brain tissue despite a long time of ischemia (usually called slow progressors) and patients in whom the ischemic brain tissue shows complete irreversible ischemic changes soon after symptom onset (fast progressors). In order to maximize the chances to observe differences in the measured outcomes between patients who will receive ApTOLL vs. placebo, patients presumed to dilute the potential treatment effect of ApTOLL are attempted to be excluded. The inclusion criteria of the APRIL study (combining time from symptom onset < 6 h and an identifiable infarct core on admission CTP) are designed to include predominantly “fast progressors” and exclude those patients in which the final infarct volume is expected to be minimal or very large with the standard treatment.

The rationale to enroll patients with moderate infarct core (5–70 cc) comes from Olivé-Gadea et al. (20). It is not infrequent that patients suffering a severe stroke that undergoes EVT will experience a dramatic clinical recovery with no residual or very little final infarct or neurological deficit. Among this group of patients, a neuroprotective drug will not be able to show its benefits and therefore including these patients in such a trial may only dilute any potential neuroprotective effect (ceiling effect). On the other hand, in those patients that will develop very large final infarcts, a potential relative reduction of final infarct size induced by the neuroprotective drug may be insufficient to induce a measurable reduction in a neurological deficit. According to this theory, patients forecasted to develop a moderate infarct size after receiving EVT would be the optimal target population in which a neuroprotectant may have the highest potential to show its clinical and radiological benefits. CTP has shown to be a valuable tool to predict on admission the final infarct volume. In particular a baseline CBF<30% showed the best correlation with final infarct volume in those patients that will achieve a significant recanalization and therefore can be considered an adequate surrogate of the infarct core on admission.

In conclusion, there is a new opportunity for neuroprotectants like ApTOLL to demonstrate their activity in combination with EVT. However, building on learning from prior treatment successes and failures, there is also a need for selecting the appropriate AIS patients' population to guarantee that the neuroprotective effect of the drug is made evident. Therefore, the APRIL study aims to replicate the promising results observed in preclinical models of transient ischemia and demonstrate the safety and efficacy of ApTOLL in the defined target population of patients with stroke treated with EVT, in whom ApTOLL has the theoretically highest chances to show positive clinical and radiological effects.

Supported by all this rationale, the APRIL trial has been designed using a novel approach to test neuroprotective drugs focusing on selecting patients with LVO and moderate infarct volumes (core between 5 and 70 cc), using appropriate image techniques (CTP and MRI) and assessing the appropriate endpoints.

The results of the APRIL trial will be published within 1 year from the last follow up of the last patient enrolled in the trial.

## Ethics statement

The studies involving human participants were reviewed and approved by Spain (REC number: 280127#), Germany (REC number: 21-9848-AF), France (REC number: 221 A01), and Portugal (REC number: 20210487). The patients/participants provided their written informed consent to participate in this study.

## Author contributions

MR and MH-J researched the literature, conceived the study, and wrote the manuscript. MH-J was involved in protocol development, gaining ethical, and regulatory approvals. MR was involved in patient recruitment and site coordination. EC, MH-J, and MR designed the statistical analysis. FA-S, IC, JG, BJ, and AF are the members of the safety committees (DSMB and CAC). MR, TJ, JV, CM, JM, JC, MD, and MH-P are members of the Steering Committee of the trial. DL and MR designed the imaging protocol. All authors reviewed and edited the manuscript and approved the final version of the manuscript.
